# Black–White Latino Racial Disparities in HIV Survival, Florida, 2000–2011

**DOI:** 10.3390/ijerph13010009

**Published:** 2015-12-22

**Authors:** Diana M. Sheehan, Mary Jo Trepka, Kristopher P. Fennie, Guillermo Prado, Miguel Ángel Cano, Lorene M. Maddox

**Affiliations:** 1 Center for Substance Use and HIV/AIDS Research on Latinos in the United States (C-SALUD) and Department of Epidemiology, Robert Stempel College of Public Health and Social Work, Florida International University, 11200 SW 8th St., Miami, FL 33199, USA; dshee004@fiu.edu; 2 Department of Epidemiology, Robert Stempel College of Public Health and Social Work, Florida International University, 11200 SW 8th St., Miami, FL 33199, USA; kfennie@fiu.edu (K.P.F.); mcanojr@fiu.edu (M.Á.C.); 3 Department of Public Health Sciences, Miller School of Medicine, University of Miami, 1120 NW 14th St., Miami, FL 33136, USA; gprado@med.miami.edu; 4 HIV/AIDS Section, Florida Department of Health, 4052 Bald Cypress Way, Tallahassee, FL 32399, USA; lorene.maddox@flhealth.gov

**Keywords:** racial disparities, Latinos, human immunodeficiency virus, mortality, neighborhood

## Abstract

This research aimed to estimate Black/White racial disparities in all-cause mortality risk among HIV-positive Latinos. Florida surveillance data for Latinos diagnosed with HIV (2000–2008) were merged with 2007–2011 American Community Survey data. Crude and adjusted hazard ratios (aHR) were calculated using multi-level Cox regression. Of 10,903 HIV-positive Latinos, 8.2% were Black and 91.9% White. Black Latinos were at increased mortality risk compared with White Latinos after controlling for individual and neighborhood factors (aHR 1.40, 95% confidence interval (CI) 1.21–1.62). In stratified analyses, risk factors for Black Latinos included: age ≥60 years compared with ages 13–19 (aHR 4.63, 95% CI 1.32–16.13); US birth compared with foreign birth (aHR 1.56, 95% CI 1.16–2.11); diagnosis of AIDS within three months of HIV diagnosis (aHR 3.53, 95% CI 2.64–4.74); residence in the 3rd (aHR 1.82, 95% CI 1.13–2.94) and 4th highest quartiles (aHR 1.79, 95% CI 1.12–2.86) of neighborhood poverty compared with the lowest quartile; and residence in neighborhood with 25%–49% (aHR 1.59, 95% CI 1.07–2.42) and ≥50% Latinos compared with <25% Latinos (aHR 1.58, 95% CI 1.03–2.42). Significant racial disparities in HIV survival exist among Latinos. Differential access to—and quality of—care and perceived/experienced racial discrimination may be possible explanations.

## 1. Introduction

Racial disparities in mortality from all causes among non-Latino populations with human immunodeficiency virus (HIV)/acquired immunodeficiency syndrome (AIDS) in the United States (US) are well documented. In 2011, the mortality rate for non-Latino Blacks with HIV was 24.2 per 1000 persons living with HIV, compared with 20.6 for non-Latino Whites [1]. Additionally, non-Latino Black males and females experience shorter life expectancy [2] and increased risk of death [3,4,5] after HIV diagnosis compared with non-Latino Whites.

Despite evidence of Black-White racial disparities in survival among non-Latino populations with HIV, national and local estimates of HIV outcomes, including survival, predominantly group Latinos of Black and White race together [1]. This common practice may be concealing significant and important disparities within Latinos. According to 2013 American Community Survey (ACS) five-year estimates, nearly 1.1 million Latinos living in the US self-identify as Black [6]. A study of 2000–2007 National Health Interview Survey data found that Black Latinos resembled White Latinos in health behaviors, but resembled non-Latino Blacks in access to care and health status [7]. In addition to experiencing worse health and health outcomes [8,9], Black Latinos also experience higher poverty, lower educational attainment, and fewer employment opportunities compared with White Latinos [10].

Identifying disparities and understanding the differences and similarities between Black and White Latinos is critical in the design—and targeting—of HIV tertiary prevention strategies. Thus, the objectives of this study were to: (a) estimate Black-White racial disparities in all-cause mortality risk among Latinos with HIV; (b) identify individual- and neighborhood-level factors contributing to racial disparities; and (c) compare individual- and neighborhood-level factors associated with mortality by race.

## 2. Data and Method

### 2.1. Datasets

De-identified HIV surveillance records from the Florida Department of Health enhanced HIV/AIDS reporting system (eHARS) were merged with administrative data from the American Community Survey [11]. Cases of Latinos age ≥13 who met the CDC HIV case definition [12] during the years 2000–2008 were included in the study. Vital status was assessed through December 2011 by linkage with Florida Vital Records, the Social Security Death Master File, and the National Death Index. Data from the 2007–2011 ACS were extracted at the ZIP code tabulation area (ZCTA)-level [11]. ZCTAs are geographic approximations of ZIP codes used to tabulate summary statistics by the US Census Bureau [13], and were used in this study to define the residential neighborhood of cases at time of HIV diagnosis. Cases with unknown or invalid data for ZIP code at time of HIV diagnosis (*n* = 700), and those diagnosed in a correctional facility (*n* = 232), were excluded.

#### Individual- and Neighborhood-Level Variables

Individual-level data extracted from eHARS included: ethnicity, race, HIV diagnosis year, sex at birth, age at HIV diagnosis, HIV transmission mode, birth country, HIV-to-AIDS diagnosis-interval in months (if case progressed to AIDS), HIV-diagnosis to death interval in months (if individual died by 31 December 2011), residential ZIP code at time of HIV diagnosis, and whether the case was diagnosed at a correctional facility. Race/ethnicity data were self-reported during HIV testing, reported by a health care provider, or extracted from medical chart reviews. Hispanic ethnicity in surveillance data is coded as “Hispanic, all races” or “Not Hispanic” with various choices for race (e.g., “Not Hispanic, Black”, “Not Hispanic, White”). No distinction is made between Hispanic and Latino ethnicity. Therefore, our definition of Latinos includes all cases reported as “Hispanic, all races”, including Latinos and Hispanics. Latinos were categorized into one of four age categories (13–19, 20–39, 40–59, 60 and older). Further categorization of those 60 and older was not possible due to the small number of older cases. Latinos were coded as US-born if they were born in any of the 50 states, District of Columbia, Puerto Rico, or any US dependent area. Cases with injection drug use (IDU) or IDU plus male-to-male sexual contact (MSM) listed as a mode of HIV transmission were categorized as having a history of IDU. IDU and MSM/IDU were combined due to the small number of cases of MSM/IDU among Black Latinos (*n* = 37). Late HIV diagnosis was defined as an AIDS diagnosis within three months of HIV diagnosis [14]. Individual-level socioeconomic data were not available.

Three neighborhood-level factors were examined: poverty, Latino ethnic density, and rural/urban status. Neighborhood-level poverty was measured using 13 socioeconomic (SES) indicators [15] extracted from the ACS: percent of households without access to a car, percent of households with ≥1 person per room, percent of population living below the poverty line, percent of owner-occupied homes worth ≥$300,000, median household income in 2011, percent of households with annual income <$15,000, percent of households with annual income ≥$150,000, income disparity (derived from percent of households with annual income <$10,000 and percent of households with annual income ≥$50,000), percent of population age ≥25 with less than a 12th grade education, percent of population age ≥25 with a graduate professional degree, percent of households living in rented housing, percent of population age ≥16 who were unemployed, and percent of population age ≥16 employed in high working class occupation (ACS occupation group: “managerial, business, science, and arts occupations”). Income disparity was calculated as the base 10 logarithm of 100 times the percent of households with annual income <$10,000 divided by the percent of households with annual income ≥$50,000 and was used as a proxy measure for the Gini-coefficient [15,16]. All income figures are presented in 2011 inflation-adjusted US dollars.

Latino ethnic density was also extracted from the ACS and defined as the percent of the population who identified as Hispanic/Latino. The ACS allows for two categories of ethnicity, “Hispanic or Latino” and “Not Hispanic or Latino”. Thus, no distinction is made between Hispanic and Latino ethnicity. Latino ethnic density was divided into three categories: <25%, 25%–49%, and ≥50% [17,18]. Rural/urban status was categorized using Categorization C of version 2.0 of the Rural-Urban Commuting Area (RUCA) codes, developed by the University of Washington WWAMI Rural Research Center [19].

### 2.2. Statistical Analysis

SAS software, version 9.4 (SAS Institute, Cary, NC 2002, USA) was used to conduct analyses. A total of 1427 (13.0%) Latino cases in our cohort were missing data on race. We created 10 datasets with imputed data for race using the PROC MI procedure in SAS. We used year of HIV diagnosis, sex, age, country of birth, and HIV transmission mode to predict race in a fully conditional specification (FCS) model [20]. All subsequent analyses were fitted by imputation by constructing pooled estimates. Following imputation, a neighborhood poverty index was developed based on the analytical methods of Niyonsenga *et al.* [15]. The 13 SES indicators were coded so that higher scores equaled higher poverty and were then standardized [15]. The reliability analysis showed a Cronbach’s alpha of 0.94 for the 13 indicators. The following seven indicators were selected based on the correlation of the indicator with the total index (high correlation), and the Cronbach’s alpha “if item deleted” (low alpha): percent below poverty, median household income, percent of households with annual income <$15,000, percent of households with annual income ≥$150,000, income disparity, percent of population age ≥25 with less than a 12th grade education, and high-class work. The Cronbach’s alpha for the seven indicators increased to 0.96. Subsequent principal component analysis with and without varimax rotation revealed one factor with an eigenvalue greater than 1 (5.56) accounting for 79.5% of the variance in the indicators. All seven indicators were retained based on high factor loadings (between 0.80 and 0.95), and were consistent with those chosen for the urban “poverty index” in the study by Niyonsenga *et al.* [15]. The standardized scores for the seven indicators were added to create a poverty index and categorized into quartiles.

Then, we compared individual- and neighborhood-level characteristics by race. We used the Cochran-Mantel-Haenszel general association statistic for individual-level variables to control for non-independence among cases within a neighborhood, and the chi-square test for neighborhood-level variables. Finally, we generated Kaplan-Meier survival curves for all-cause mortality by race. The Cox proportional hazards assumption was tested and met. Multi-level (level 1: individual; level 2: neighborhood) models were used to account for correlation among cases living in the same neighborhood. Crude and adjusted hazard ratios and 95% confidence intervals were calculated comparing Black Latinos with White Latinos using PROC PHREG. Hazard ratios were adjusted for year of HIV diagnosis, sex, age, U.S./foreign-born status, mode of HIV transmission, late HIV diagnosis, neighborhood poverty, Latino ethnic density, and rural/urban status. Analyses were stratified by race to identify predictors of mortality for each group. The Florida International University institutional review board approved this study and the Florida Department of Health designated this study to be non-human subjects research.

## 3. Results

### 3.1. Characteristics of Cases

Of 10,989 Latinos diagnosed with HIV in Florida between 2000 and 2008, 792 (7.2%) were reportedly Black; 8712 (79.3%) White; 58 (0.5%) other; and 1427 (13.0%) unknown race. After imputation, there were only 86 cases of races other than Black or White and therefore they were excluded from further analyses. Analyses with imputed data for race included 10,903 Latinos: 889 (8.2%) Black Latinos and 10,014 (91.9%) White Latinos (Table 1). The proportions that were female (30.7%) and US-born (52.5%) were significantly higher for Black Latinos than for White Latinos (19.7% and 39.0%, respectively). For Black Latinos, infections were predominantly attributed to MSM and heterosexual contact; for White Latinos, over half of infections were attributed to MSM. About one third (34%) of infections among Blacks and Whites were diagnosed late, and almost all individuals (98%)—Blacks and Whites alike—lived in urban neighborhoods. A larger proportion of Black Latinos lived in the highest quartile of neighborhood poverty (34.7%) compared with White Latinos (25.1%). The proportion of Black Latinos that lived in neighborhoods with ≥50% Latino ethnic density was smaller (28.6%) compared with White Latinos (38.2%).

**Table 1:** Characteristics of Black and White race Latinos aged 13 years and older reported with HIV, Florida, 2000–2008.

Characteristic	Race			
Total	Total, *n* 10,903	Black	White	*p*-Value ^b^
		*n* (%)	*n* (%)	
		889 (8.2)	10,014 (91.9)	
**Individual-level variables**				
Year of HIV diagnosis				<0.0001
2000–2002	3793 (34.8)	385 (43.3)	3408 (34.0)	
2003–2005	3640 (33.4)	266 (29.9)	3374 (33.7)	
2006–2008	3470 (31.8)	238 (26.8)	3232 (32.3)	
Sex at birth				<0.0001
Male	8661 (79.4)	616 (69.3)	8045 (80.3)	
Female	2242 (20.6)	273 (30.7)	1969 (19.7)	
Age group at diagnosis				0.0971
13–19 years	238 (2.2)	34 (3.8)	204 (2.0)	
20–39 years	5938 (54.5)	464 (52.2)	5474 (54.7)	
40–59 years	4204 (38.6)	349 (39.3)	3855 (38.5)	
60 years or older	523 (4.8)	42 (4.7)	481 (4.8)	
US- *vs.* foreign-born				<0.0001
US-born **^c^**	4374 (40.1)	467 (52.5)	3908 (39.0)	
Foreign-born	6529 (59.9)	422 (47.5)	6106 (61.0)	
Mode of HIV transmission				<0.0001
Male-to-male sexual				
contact (MSM)	5668 (52.0)	317 (35.7)	5351 (53.4)	
Injection drug use (IDU) **^a^**	1120 (10.3)	110 (12.4)	1010 (10.1)	
Heterosexual contact	2798 (25.7)	352 (39.6)	2446 (24.4)	
Other/unknown	1317 (12.1)	110 (12.4)	1207 (12.1)	
Late HIV diagnosis (AIDS diagnosis within 3 months of HIV diagnosis)				0.2800
Yes	3483 (32.0)	304 (34.2)	3179 (31.8)	
No	7420 (68.1)	585 (65.8)	6835 (68.3)	
Three-year survival				0.0042
Yes (alive)	9836 (90.2)	768 (86.4)	9068 (90.6)	
No	1067 (9.8)	121 (13.6)	946 (9.5)	
Five-year survival				0.0005
Yes (alive)	9571 (87.8)	736 (82.8)	8835 (88.2)	
No	1332 (12.2)	153 (17.2)	1179 (11.8)	
**ZCTA-level variables**				
Poverty index, quartiles **^d^**				<0.0001
1 (lowest poverty)	2746 (25.2)	191 (21.5)	2555 (25.5)	
2	2716 (24.9)	171 (19.2)	2545 (25.4)	
3	2619 (24.0)	219 (24.6)	2400 (24.0)	
4 (highest poverty)	2822 (25.9)	308 (34.7)	2514 (25.1)	
Latino ethnic density (percent of population who self-identified as Hispanic/Latino)				<0.0001
≥50	4081 (37.4)	254 (28.6)	3827 (38.2)	
25–49	3287 (30.2)	262 (29.5)	3025 (30.2)	
<25	3535 (32.4)	373 (42.0)	3162 (31.6)	
RUCA classification				
Rural	215 (2.0)	19 (2.1)	196 (2.0)	0.7115
Urban	10,688 (98.0)	870 (97.9)	9818 (98.0)	

Notes: U.S. = United States; ZCTA = ZIP code tabulation area; RUCA = Rural-Urban Commuting Area. Missing race data were imputed using year of HIV diagnosis, sex, age, country of birth, and HIV transmission mode. Excludes cases diagnosed in a correctional facility (*n* = 232), missing residential zip code at time of HIV diagnosis (*n* = 700), or diagnosed under the age of 13 (*n* = 32). Percentages may not add up to 100 due to rounding. **^a^** Includes cases reported as injection drug use only (*n* = 808; Black Latino *n* = 73; White Latino *n* = 735) or both male-to-male sexual contact and injection drug use (*n* = 312; Black Latino *n* = 37; White Latino *n* = 275); **^b^** *p*-Value for individual-level variables from Cochran-Mantel-Haenszel test controlling for residential zip code. *p*-Value for neighborhood-level variables from chi-square test; **^c^** Category includes cases born in any of the 50 US states and District of Columbia (*n* = 3268; Black Latino *n* = 378; White Latino *n* = 2890), Puerto Rico (*n* = 1095; Black Latino *n* = 87; White Latino *n* = 1008), or any US dependency (*n* = 11; Black Latino *n* = 2; White Latino *n* = 9); **^d^** SES quartiles of standardized SES scores of all Latino cases in eHARS.

### 3.2. Black-White Latino Racial Disparities in All-Cause Mortality

**Figure 1:**
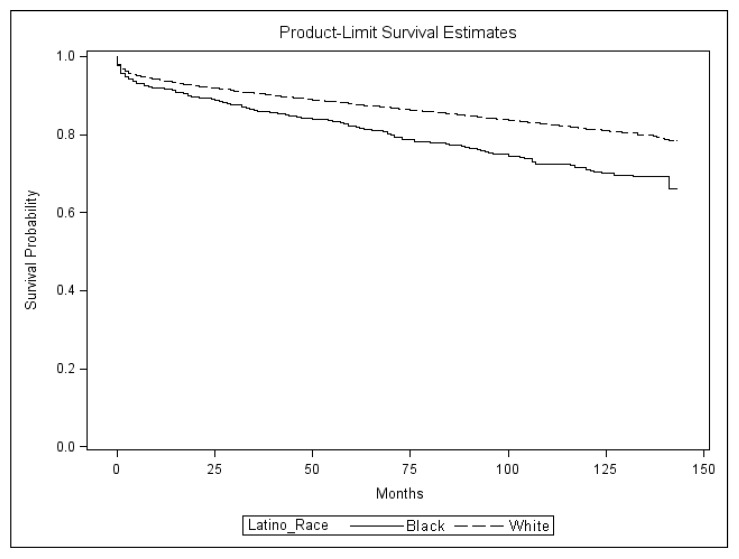
Survival after HIV diagnosis probability curves for Latinos 13 years of age and older reported with HIV by race, Florida, 2000–2011. Includes imputed data for race. Curves are unadjusted for age.

The proportion of Black Latinos alive at 3- (86.4%) and 5-years (82.8%) after HIV diagnosis was significantly lower compared with White Latinos (90.6% and 88.2%, respectively) (Table 1; Figure 1). The crude mortality hazard ratio for all-cause mortality for Black Latinos was 1.59 that of White Latinos (95% confidence interval (CI) 1.38–1.83) (Table 2). The hazard ratio decreased to 1.46 (95% CI 1.26–1.70) after controlling for individual-level factors, and to 1.40 (95% CI 1.21–1.62) after controlling for both individual- and neighborhood-level factors. After adjusting for individual- and neighborhood-level variables, the hazard ratio for Black US-born compared with White US-born Latinos was 1.57 (95% CI 1.29–1.92). Among foreign-born Latinos, the hazard ratio for Black compared with White Latinos was 1.28 after controlling for individual- and neighborhood-level factors (95% CI 1.03–1.59). Among all deaths, 68.5% had an underlying cause of death listed in the death certificate of HIV. The proportion of deaths with an underlying cause of death due to HIV was greater for Black Latinos (72.8%) compared with White Latinos (67.8%), but not statistically significant (*p*-value 0.1777).

### 3.3. Predictors of All-Cause Mortality by Race

Predictors of all-cause mortality differed by race among Latinos. Increased hazard ratios were present in Black Latinos who: were 60 years of age or older at time of HIV diagnosis compared with age 13–19 (adjusted hazard ratio (aHR) 4.63, 95% CI 1.32–16.13), were US-born compared with foreign-born (aHR 1.56, 95% CI 1.16–2.11), reported mode of HIV transmission of other or unknown compared with heterosexual contact (aHR 1.63, 95% CI 1.08–2.45), were diagnosed with HIV late (aHR 3.53, 95% CI 2.64–4.74), lived in the 3rd and 4th highest quartiles of neighborhood poverty compared with the lowest quartile (3rd quartile aHR 1.82, 95% CI 1.13–2.94; 4th quartile aHR 1.79, 95% CI 1.12–2.86), and lived in areas with 25%–49% (aHR 1.59, 95% CI 1.07–2.42) and ≥50% Latino ethnic density (aHR 1.58, 95% CI 1.03–2.42) (Table 3). Additional risk factors for White Latinos included male compared with female sex (aHR 1.26, 95% CI 1.08–1.47), being 40–59 years of age compared with age 13–19 (aHR 3.03, 95% CI 1.65–5.56), and a reported mode of HIV transmission of injection drug use (aHR 1.74, 95% CI 1.47–2.07). There were no significant differences in hazard of mortality by Latino ethnic density among White Latinos.

**Table 2:** Crude and adjusted hazard ratios and 95% confidence intervals for all-cause mortality among Black and White Latinos 13 years of age and older reported with HIV, Florida, 2000–2011.

Race	Model 1 Crude HR (95% CI)	Model 2 Adjusted HR (95% CI)	Model 3 Adjusted HR (95% CI)
**All Latinos**			
Black	1.59 (1.38–1.83)	1.46 (1.26–1.70)	1.40 (1.21–1.62)
White	Referent	Referent	Referent
**U.S.-born Latinos**			
Black	1.54 (1.27–1.86)	1.64 (1.34–2.00)	1.57 (1.29–1.92)
White	Referent	Referent	Referent
**Foreign-born Latinos**			
Black	1.57 (1.27–1.94)	1.29 (1.03–1.61)	1.28 (1.03–1.59)
White	Referent	Referent	Referent

Notes: HR, hazard ratio for mortality; CI, confidence interval. Includes imputed data for race. Model 1: Includes race only; Model 2: Includes race and individual-level variables (year of HIV diagnosis, sex at birth, age, U.S./foreign born, mode of HIV transmission, and late HIV diagnosis); Model 3: Includes race, individual-level variables, and neighborhood-level variables (poverty, Latino ethnic density, and rural/urban status).

## 4. Discussion

The primary finding of this study is that Black Latinos diagnosed with HIV in Florida between 2000 and 2008 had an increased hazard of all-cause mortality compared with White Latinos with HIV, even after controlling for individual- and neighborhood-level factors. Findings suggest that racial disparities were stronger among US-born Latinos. Additionally, predictors of mortality differed by race. Risk factors for mortality among Black Latinos included being 60 years of age or older, U.S.-born, and diagnosed late, as well as living in a neighborhood of high poverty and high Latino ethnic density. Mortality for White Latinos was additionally associated with being male, and reporting a history of injection drug use, but was not associated with neighborhood Latino ethnic density.

**Table 3:** Crude and adjusted hazard ratios and 95% confidence intervals for all-cause mortality among Black and White Latinos 13 years and older reported with HIV, Florida, 2000–2011.

Characteristic	Race			
	Black		White	
	Crude HR (95% CI)	Adjusted HR (95% CI)	Crude HR (95% CI)	Adjusted HR (95% CI)
Individual-level variables				
Year of HIV diagnosis				
2000–2002	1.44 (0.95–2.18)	1.29 (0.84–1.99)	1.42 (1.24–1.63)	1.33 (1.16–1.54)
2003–2005	1.45 (0.93–2.26)	1.22 (0.78–1.92)	1.19 (1.03–1.37)	1.10 (0.95–1.27)
2006–2008	Referent	Referent	Referent	Referent
Sex at birth				
Male	0.98 (0.74–1.30)	0.93 (0.65–1.33)	1.12 (0.99–1.28)	1.26 (1.08–1.47)
Female	Referent	Referent	Referent	Referent
Age group at diagnosis				
13–19 years	Referent	Referent	Referent	Referent
20–39 years	1.82 (0.59–5.68)	1.37 (0.42–4.42)	1.99 (1.07–3.68)	1.57 (0.86–2.88)
40–59 years	4.03 (1.31–12.05)	2.83 (0.88–9.09)	4.63 (2.51–8.55)	3.03 (1.65–5.56)
60 years or older	6.94 (2.10–23.26)	4.63 (1.32–16.13)	12.05 (6.49–22.73)	7.94 (4.29–14.71)
US- *vs.* foreign-born				
US-born	1.15 (0.88–1.50)	1.56 (1.16–2.11)	1.17 (1.06–1.30)	1.23 (1.10–1.37)
Foreign-born	Referent	Referent	Referent	Referent
Mode of HIV transmission				
Male-to-male sexual contact (MSM)	0.95 (0.69–1.31)	1.09 (0.74–1.63)	0.77 (0.68–0.87)	0.87 (0.74–1.01)
Injection drug use (IDU)	1.04 (0.68–1.58)	0.82 (0.50–1.33)	1.90 (1.63–2.21)	1.74 (1.47–2.07)
Heterosexual contact	Referent	Referent	Referent	Referent
Other/unknown	1.94 (1.31–2.87)	1.63 (1.08–2.45)	1.63 (1.39–1.92)	1.51 (1.27–1.78)
Late HIV diagnosis (AIDS diagnosis within 3 months of HIV diagnosis)				
Yes	3.36 (2.56–4.39)	3.53 (2.64–4.74)	3.24 (2.93–3.58)	2.88 (2.60–3.19)
No	Referent	Referent	Referent	Referent
ZCTA-level variables				
Poverty index, quartiles				
1 (lowest poverty)	Referent	Referent	Referent	Referent
2	1.04 (0.63–1.72)	1.14 (0.66–1.97)	1.02 (0.85–1.24)	1.01 (0.84–1.21)
3	1.54 (0.99–2.39)	1.82 (1.13–2.94)	1.29 (1.08–1.53)	1.24 (1.05–1.47)
4 (highest poverty)	1.80 (1.18–2.74)	1.79 (1.12–2.86)	1.62 (1.35–1.94)	1.51 (1.27–1.80)
Latino ethnic density (percent of population who self-identified as Hispanic/Latino)				
≥50	1.49 (1.04–2.13)	1.58 (1.03–2.42)	1.02 (0.85–1.22)	1.10 (0.94–1.28)
25–49	1.66 (1.18–2.34)	1.59 (1.07–2.42)	1.05 (0.88–1.25)	1.09 (0.93–1.27)
<25	Referent	Referent	Referent	Referent
RUCA classification				
Rural	0.95 (0.38–2.36)	0.90 (0.34–2.41)	1.70 (1.23–2.35)	1.28 (0.93–1.76)
Urban	Referent	Referent	Referent	Referent

Notes: HR, hazard ratio for mortality; CI, confidence interval. Includes imputed data for race.

The finding that Black Latinos with HIV are at increased mortality risk compared with White Latinos with HIV is important. The disparity not only remains after controlling for individual- and neighborhood-level factors, but it also appears to be at least as great as the racial disparity in mortality between Black and White non-Latinos with HIV [3,4,5]. Two studies, including one in Florida, found that survival disparities between non-Latino Blacks and non-Latino Whites with AIDS disappeared after controlling for neighborhood poverty [3,5]. Our study differed in that—in addition to AIDS cases—we also examined individuals with HIV; however, it is noteworthy that racial disparities among Latinos in our study remained after controlling for a comprehensive measure of neighborhood poverty. Our findings also suggest that racial disparities were not due to differences in late HIV diagnosis (*i.e.*, differences in HIV testing), but were more likely due to differences in access to and quality of HIV care and/or HIV medication adherence.

LaVeist-Ramos *et al.* suggest that Latino ethnicity may affect health behaviors through cultural values and beliefs [7]. Societal forces, such as discrimination, may affect health status and access to care differentially by race [7]. In their study, LaVeist-Ramos and colleagues found that Black Latinos’ health behaviors were similar to that of White Latinos, but services outcomes were similar to that of non-Latino Blacks. Perceived Latino ethnic discrimination has been shown to decrease utilization [21] and quality of care [22], and be associated with negative health outcomes among Latinos [23,24,25]. Black Latinos may additionally experience racial discrimination. Perez-Escamilla suggests that Black Latinos in the US “have a double burden of perceived racial discrimination associated with being Black and Latino” ([26], Page 1166S). Among HIV-positive Latinos, perceived ethnic and racial discrimination is compounded with HIV-related discrimination [27], and for some, discrimination related to sexual minority status [28,29] and/or drug use [30].

In our study, the mortality hazard ratio between Black and White Latinos increased for US-born Latinos and decreased among foreign-born Latinos after controlling for individual- and neighborhood-level factors. It is possible that racial differences in access to and quality of care are only present, or more pronounced, among US-born Latinos. Foreign-born Latinos experience different access to and utilization of care barriers, such as English language proficiency [31] and immigrant documentation status [32], that are unrelated to race. Individual-level socioeconomic status may also differ between U.S.- and foreign-born Latinos, variables that were not available in this study. Additionally, research suggests that Latinos who are second-generation immigrants, U.S.-born, and those who arrived to the US at younger ages are more likely to perceive discrimination compared with first-generation Latinos and Latinos arriving at older ages [33,34]. It is possible that discrimination was more prevalent among US-born Black Latinos compared with foreign-born Black Latinos, thus creating a disparity in health and health care between the groups. Additionally, first- and second-generation Latinos may perceive, report, and attribute experiences to discrimination differently.

Individual-level predictors of mortality among Latinos in our study differed by race. White Latino males were at increased mortality risk compared with White Latino females. There were no gender differences among Black Latinos. Similarly, Latinos with HIV attributable to injection drug use were at increased mortality risk compared with Latinos with HIV attributable to heterosexual contact among White Latinos only. It is possible that the small number of Black Latinos with a history of injection drug use (*n* = 71) limited our ability to find an association in this group. Late HIV diagnosis appears to be a stronger predictor of mortality for Black Latinos than for White Latinos.

Neighborhood-level determinants of mortality also differed by race. Residing in a neighborhood in the highest two quartiles of neighborhood poverty compared with the lowest quartile increased mortality risk for both White and Black Latinos with HIV. However, among Latinos with HIV, the effect of poverty appeared to be stronger for Blacks than for Whites. Economic opportunity among Black Latinos may be further diminished in disadvantaged areas compared with White Latinos. Wage discrimination has been documented among Black Latinos, above and beyond that experienced by non-Latino Blacks [35]. Black Latinos who resided in neighborhoods with 25%–49% and ≥50% Latino ethnic density were at increased risk of mortality compared with Black Latinos who resided in neighborhoods with <25% Latino ethnic density. This finding was not observed among White Latinos and is inconsistent with the main body of literature, which shows that higher Latino ethnic density is protective for all-cause mortality among the general Latino population [36]. However, previous studies have not reported results stratified by race for Latinos. Therefore, it is unclear how Black Latinos, specifically, fare in a predominantly Latino neighborhood. Black Latinos perceive discrimination not only from non-Latinos, but from White Latinos as well. Research across countries in Central and South America and the Caribbean show a preference among Latinos for lighter skin and White phenotype compared with darker skin or Black phenotype [37]. Black Latinos are not only an ethnic minority, but also a racial minority within (and outside) their ethnic group. However, levels of racial discrimination may vary widely by country and region of Latin America. In addition, studies of Latino ethnic density have generally examined non-stigmatizing conditions [18,36,38]. Findings from the present study and two other recent studies of HIV-positive Latinos [39,40] suggest that living within a predominantly Latino community is unfavorable for Latinos with HIV. High levels of HIV/AIDS-related stigma [41] and low levels of HIV/AIDS knowledge [42] in the Latino community may be a possible explanation by removing otherwise available social support. Low levels of HIV disclosure among Latinos with HIV [43] may also permit stigma to remain and social support in high Latino density neighborhoods [44,45,46] for Latinos with HIV to remain low.

The main limitation of our study is that self-reported and medical records data on race among Latinos may be imprecise. Findings from a study conducted by the Pew Research Center suggest that Latinos may not see themselves as fitting into racial categories [47]. This is reflected in the 26% of Latinos who reported “some other race” in the 2013 American Community Survey [48]. In our study, 13% of Latinos diagnosed between 2000 and 2008 were listed as unknown race. Our imputation method may have introduced bias if race was related to un-observed factors not included in the imputation model. However, we ran the analysis excluding those with missing race and found the same associations. The manner in which race data are collected for HIV surveillance may also pose challenges as the data may be self-reported, or assigned by a physician or laboratory technician. A study in a clinic setting found that up to 33% of Latinos held racial self-perceptions that differed from the racial categorizations noted in their medical records [49]. Discrepancies in recorded *vs.* perceived race may introduce significant complexity in the interpretation of our results, particularly when discussing perceived racial discrimination and access to and quality of care. Misclassification of race may occur in data reported by health care providers.

Additionally, we were unable to determine ethnic origin for US-born Latinos. It is possible that ethnic origin differed between US- and foreign-born Latinos, and that these cultural differences partially explain why racial disparities remained only among those born in the US. We do know, however, that 18.5% of Black US-born Latinos were born in Puerto Rico, a group that experiences shorter survival after HIV diagnosis compared with Latinos born in mainland US and foreign countries [50] and report the highest levels of ethnic discrimination among Latinos [33,51]. We were unable to examine individuals born in Puerto Rico (or other U.S. dependencies) separately due to the small number of Black Latinos born in Puerto Rico (*n* = 87). Nevertheless, among all Latinos in our cohort, those born in Puerto Rico were more likely to be older and of White race, report IDU, and be diagnosed late, and less likely to be alive at 3- and 5-years after HIV diagnosis compared with those born in the 50 U.S. states. Foreign-born Black Latinos were born mainly in Cuba (28%), the Dominican Republic (11%), and Honduras (9%), and 10% had missing information for birth country. Cubans report the lowest levels of ethnic discrimination among Latinos [33,51]. Our study was also limited to general data collected for HIV/AIDS surveillance purposes. Therefore, we were unable to control for variables such as individual-level socioeconomic status, length of time in the US, immigrant documentation status, and health insurance status. Finally, the Florida Latino population differs in ethnic origin from the U.S. Latino population—possibly restricting the generalizability of our study.

Regarding our analysis of neighborhood characteristics, there are three main limitations. First, we were limited to using ZIP codes to define neighborhoods. Although smaller geographic units (e.g., block groups) may be more precise, ZIP codes have been shown to provide valid results for all-cause mortality [52]. Second, the use of a poverty index may limit comparison of results to studies using a single measure of neighborhood poverty [52,53]. Still, our poverty index was highly correlated with the percent of the population under the poverty level—a commonly used single variable measure of neighborhood poverty (Pearson correlation 0.91). Lastly, we had relatively complete data on ZIP code of residence at time of HIV diagnosis, but incomplete data on ZIP code throughout the follow-up period, or at time of death. Therefore, our study is not able to determine the length of time that cases were exposed to neighborhood characteristics.

## 5. Conclusions

A distinction between ethnicity and race among Latinos in research has been proposed [54,55]; however, few major studies of Latinos have incorporated race. To our knowledge this is the first population-based cohort study examining racial disparities in mortality risk among Latinos with HIV, and the associated individual- and neighborhood-level factors. The findings of our study highlight a relatively large Black-White racial disparity in all-cause mortality risk among Latinos with HIV. Furthermore, it suggests that factors beyond age, HIV transmission mode, late HIV diagnosis, and neighborhood poverty and ethnic composition contribute to racial disparities in this population. Our study also suggests that tertiary prevention interventions for HIV-positive Latinos need to be tailored to address risk and protective factors specific to Black and White Latinos. Future studies are needed to uncover the mechanisms contributing to racial disparities in mortality among Latinos with HIV, including access to and quality of care, as well as to understand the role of multiple and compounded routes of perceived and experienced discrimination. Mediators between racial discrimination and HIV outcomes, such as depression, should also be explored [56].
